# Limitations of SpO_2_ / FiO_2_-ratio for classification and monitoring of acute respiratory distress syndrome—an observational cohort study

**DOI:** 10.1186/s13054-025-05317-7

**Published:** 2025-02-19

**Authors:** Rolf Erlebach, Una Pale, Tilman Beck, Sasa Markovic, Marko Seric, Sascha David, Emanuela Keller

**Affiliations:** 1 Institute of Intensive Care Medicine, University Hospital Zurich and University of Zurich, Zurich, Switzerland; 2 Neurocritical Care Unit, Department of Neurosurgery and Institute of Intensive Care Medicine, University Hospital Zurich and University of Zurich, Zurich, Switzerland

**Keywords:** Acute hypoxemic respiratory failure, Pulse oximetry, SF ratio, PaO_2_ / FiO_2_ ratio, Data analysis

## Abstract

**Background:**

The ratio of pulse-oximetric peripheral oxygen saturation to fraction of inspired oxygen (SpO_2_/FiO_2_) has been proposed as additional hypoxemia criterion in a new global definition of acute respiratory distress syndrome (ARDS). This study aims to evaluate the clinical and theoretical limitations of the SpO_2_/FiO_2_-ratio when using it to classify patients with ARDS and to follow disease progression.

**Methods:**

Observational cohort study of ARDS patients from three high-resolution Intensive Care Unit databases, including our own database ICU Cockpit, MIMIC-IV (Version 3.0) and SICdb (Version 1.0.6). Patients with ARDS were identified based on the Berlin criteria or ICD 9/10-codes. Time-matched datapoints of SpO_2_, FiO_2_ and partial pressure of oxygen in arterial blood (PaO_2_) were created. Severity classification followed the thresholds for SpO_2_/FiO_2_ and PaO_2_/FiO_2_ of the newly proposed global definition.

**Results:**

Overall, 708 ARDS patients were included in the analysis. ARDS severity was misclassified by SpO_2_/FiO_2_ in 33% of datapoints, out of which 84% were classified as more severe. This can be partially explained by imprecision of SpO_2_ measurement and equation used to transform SpO_2_/FiO_2_ to PaO_2_/FiO_2._ A high dependence of SpO_2_/FiO_2_-ratio on FiO_2_ settings was found, leading to major treatment effect and limited capability for tracking change in ARDS severity, which was achieved in less than 20% of events.

**Conclusions:**

The use of SpO_2_/FiO_2_ interchangeably with PaO_2_/FiO_2_ for severity classification and monitoring of ARDS is limited by its inadequate trending ability and high dependence on FiO_2_ settings, which may influence treatment decisions and patient selection in clinical trials.

**Supplementary Information:**

The online version contains supplementary material available at 10.1186/s13054-025-05317-7.

## Background

Current and former definitions of acute respiratory distress syndrome (ARDS) include a hypoxemia criterion [1–3] for diagnosis and severity classification. Traditionally, this has been assessed by the ratio of partial pressure of oxygen in arterial blood (PaO_2_) divided by the fraction of inspired oxygen (FiO_2_) [3], which was used by major interventional trials in ARDS [4–6]. Because of its simplicity and practicality, the PaO_2_/FiO_2-_ratio is the most widely used surrogate of oxygen transfer in the lungs to follow disease progression and response to therapy in patients with and without ARDS [7]. The main disadvantage of PaO_2_/FiO_2_-ratio is the need of invasive arterial blood gas (ABG) sampling, which is only available intermittently, may not be readily available in resource-limited regions and is associated with some complications, such as vascular injury, hematoma, infection, thrombosis and nerve injury [8]. There is increasing interest of a continuous, non-invasive surrogate for severity classification and monitoring of ARDS [9], such as the peripheral oxygen saturation (SpO_2_) divided by FiO_2_ (SpO_2_/FiO_2_) ratio. The SpO_2_/FiO_2_-ratio as a surrogate of PaO_2_/FiO_2_-ratio has been evaluated, showing good performance as long as SpO_2_ is ≤ 97% [10–14]. Recently, a new global definition of ARDS [15] added the SpO_2_/FiO_2_-ratio in an effort to provide an ARDS definition that is more suitable for resource-limited settings. Despite, that ARDS patients.

diagnosed with the SpO_2_/FiO_2_-ratio, seem to have similar outcomes compared to patients diagnosed with the PaO_2_/FiO_2_-ratio [16], these scores may not be used in parallel [17].

We hypothesized, that the SpO_2_/FiO_2_-ratio can be used as a surrogate for PaO2/FiO2 and that classification of ARDS is similar with both ratios. To assess the limitations of the SpO_2_/FiO_2_-ratio as a surrogate for PaO_2_/FiO_2_-ratio in the classification of ARDS severity, we focused on classification accuracy and monitoring of disease progression using retrospective data from three high-resolution ICU databases. Additionally, we provide theoretical and technical explanations for classification discrepancies.

## Methods

### Study design and datasets

This is a retrospective observational cohort study of ARDS patients using data from three high-resolution ICU databases: ICU Cockpit [18], Medical Information Mart for Intensive Care (MIMIC)-IV (version 3.0) [19, 20] and SICdb (version 1.0.6) [21, 22].

MIMIC-IV, is a large deidentified dataset of patients admitted to the Beth Israel Deaconess Medical Center in Boston (USA), between 2008 and 2022. It contains comprehensive information for over 65,000 patients admitted to an intensive care unit (ICU), including free-text radiology reports. Most of the continuous signals are provided in a resolution of one hour.

The SICdb database is a deidentified medical records dataset of patient admitted to the ICU at the University Hospital Salzburg (Austria), between 2013 and 2021 and contains vital signs, laboratory results, medication data and admission details for over 27,000 ICU admissions. SICdb provides continuous time signals with highly granular once-per-minute data.

ICU cockpit is a big data platform collecting high-resolution (up to 200 Hz for continuous signals) multimodal data since 2016, including vital signs, ventilator settings and measurements, neuromonitoring data, laboratory analyses and clinical annotations. The platform includes over 2400 patients from the Neurocritical Care Unit and the medical ICU of the University Hospital Zurich (Switzerland).

The collection and usage of data from ICU cockpit was approved by the local ethics commission (Cantonal Ethics Commission of Zurich, BASEC Nr. PB 2016–01101). Access to MIMIC-IV and SICdb was provided through PhysioNet [23] after a credential process including training and signing a data use agreement.

### ARDS population

Adult patients with ARDS were selected in the ICU Cockpit database by manually applying the Berlin definition [3]. Patients in the external databases SICdb and MIMIC-IV were identified by ICD codes (ICD-9: 51,882, ICD-10: J80). The hypoxemia criterion was modified for all databases to include patients with PaO_2_/FiO_2_ ≤ 300 mmHg measured during invasive mechanical ventilation, non-invasive ventilation (NIV) or continuous positive airway pressure (CPAP) with positive end-expiratory pressure (PEEP) ≥ 5 cmH_2_O or during high-flow nasal oxygen (HFNO) with flow ≥ 30 L/min. If patients were admitted multiple times to the ICU during one hospital stay, only the first ICU admission was kept, as subsequent ICU admissions likely represent complicated progression of disease, rather than newly developed ARDS. Patients that were supported by extracorporeal membrane oxygenation were excluded from analysis.

Because ARDS diagnosis is frequently missed by clinicians [24], the analysis was repeated in an extended population of the MIMIC-IV database by manually selecting ICU admissions based on ICU chart data, ABG results, chest radiography notes and/or ICD codes.

### Variables and data management

For each time point of ABG measurement a set of variables containing: PaO_2_, FiO_2_ and SpO_2_ was created (subsequently referred to as datapoints). For MIMIC-IV, whose data was mainly on hourly resolution, matching between FiO_2_, SpO_2_ and PaO_2_ was performed with a resolution of 30 min. In ICU Cockpit the correct time point of ABG sampling was identified with arterial pressure waveform analysis using gaps in pressure readings. When gap in pressure data was not located within 15 min prior to ABG analysis timestamp, median value between 5 and 2 min before ABG time stamp was calculated both for SpO_2_ and FiO_2_ and matched with PaO_2_ values to form datapoints. In SICdb, time delay allowed for FiO_2_ (and SpO_2_) and PaO_2_ matching was 5 min too. Datapoints were considered valid if SpO_2_ was ≤ 97% and measurements were taken during invasive mechanical ventilation/NIV/CPAP with PEEP ≥ 5 cmH_2_O or HFNO with flow ≥ 30 L/min. Incomplete sets of variables were excluded from analysis.

ARDS severity was calculated with the PaO_2_/FiO_2_-ratio and the SpO_2_/FiO_2_-ratio. Additional, most severe category per ICU admission was calculated requiring at least two consecutive classifications into an equally or more severe category. Thresholds for severity categories followed the new global definition [15] (Table 1).

**Table 1:** ARDS severity classifications according to the 2024 Global Definition [15]

	PaO_2_/FiO_2_	SpO_2_/FiO_2_
No	> 300 mmHg	> 315
Mild	> 200 mmHg and ≤ 300 mmHg	> 235 and ≤ 315
Moderate	> 100 mmHg and ≤ 200 mmHg	> 148 and ≤ 235
Severe	≤ 100 mmHg	≤ 148

### Analysis

Clinical performance of the SpO_2_/FiO_2_-ratio was evaluated on a datapoint and an ICU admission level using overall accuracy (correct classifications divided by total classifications) as a primary outcome. We further analyzed the following secondary outcomes. Accuracy per severity category was visualized using confusion matrices. Impact of FiO_2_ settings on ARDS severity category, was assessed by density plots of FiO_2_ and through analysis of accuracy per FiO_2_ values (grouped by 5%). We evaluated trending ability by correlating corresponding changes in FiO_2,_ SpO_2_ and PaO_2_ over two consecutive datapoints in the same patient. For assessing the effect of FiO_2_ changes over time, the effect of true changes in oxygenation capacity between consecutive datapoints was reduced by excluding datapoint-pairs with time-interval > 6 h and changes in respiratory index > 20%. The respiratory index (RI) [25] is a tension-based index of oxygenation, calculated as A-a gradient [26] divided by PaO_2_ and was shown to be superior to other indices of oxygenation regarding variability with different FiO2 values [27].

Limitations of SpO_2_ measurements were evaluated through comparison with arterial oxygen saturation (SaO_2_) from ABG, calculating bias and precision. A Bland–Altman plot was not used, because of a heteroscedastic bias leading to misinterpretation of the limits of agreement. Finally, conversion between PaO_2_/FiO_2_ and SpO_2_/FiO_2_ was evaluated by comparing our data and published linear [10–12] and log-linear [13, 14] imputations using performance metrics R^2^ and mean absolute error.

In the paper, we report results for three datasets combined, while analogous results for the extended MIMIC-IV population are shown in the Supplementary Information Figs. S7–S9, to address a possible selection bias introduced by using the ICD codes.

All the analysis and graphical output was performed using Python version 3.12.2.

## Results

### Population

Combining three databases led to the identification of 11,916 valid datapoints representing 708 ICU admissions. While MIMIC-IV had the highest number of admissions, data resolution was higher in ICU-Cockpit and SICdb (Table 2). Flowcharts with detailed results of the selection process are provided in the Supplementary Information (see Supplementary Information Figs. S1 and S2).

**Table 2:** Number of admissions and datapoints per database

	Overall	ICU-Cockpit	SICdb	MIMIC-IV
Admissions, n (%)	708	16 (2.3)	24 (3.4)	668 (94.3)
Datapoints, n (%)	11,916	941 (7.9)	1790 (15.0)	9185 (77.1)
Datapoints per admission, mean ± SD	16.9 ± 26.1	58.8 ± 61.2	74.6 ± 84.8	13.8 + 14.7

Based on the PaO_2_/FiO_2_-ratio, datapoints were classified as no, mild, moderate, and severe ARDS in 3.2%, 15.6%, 61.7%, 19.6%. On an admission level 7.9%, 7.2%, 47.0% and 37.9% patients were classified as no, mild, moderate, and severe ARDS (see Supplementary Information Fig. S3).

### Clinical performance of the SpO_2_/FiO_2_-ratio

**Fig. 1:**
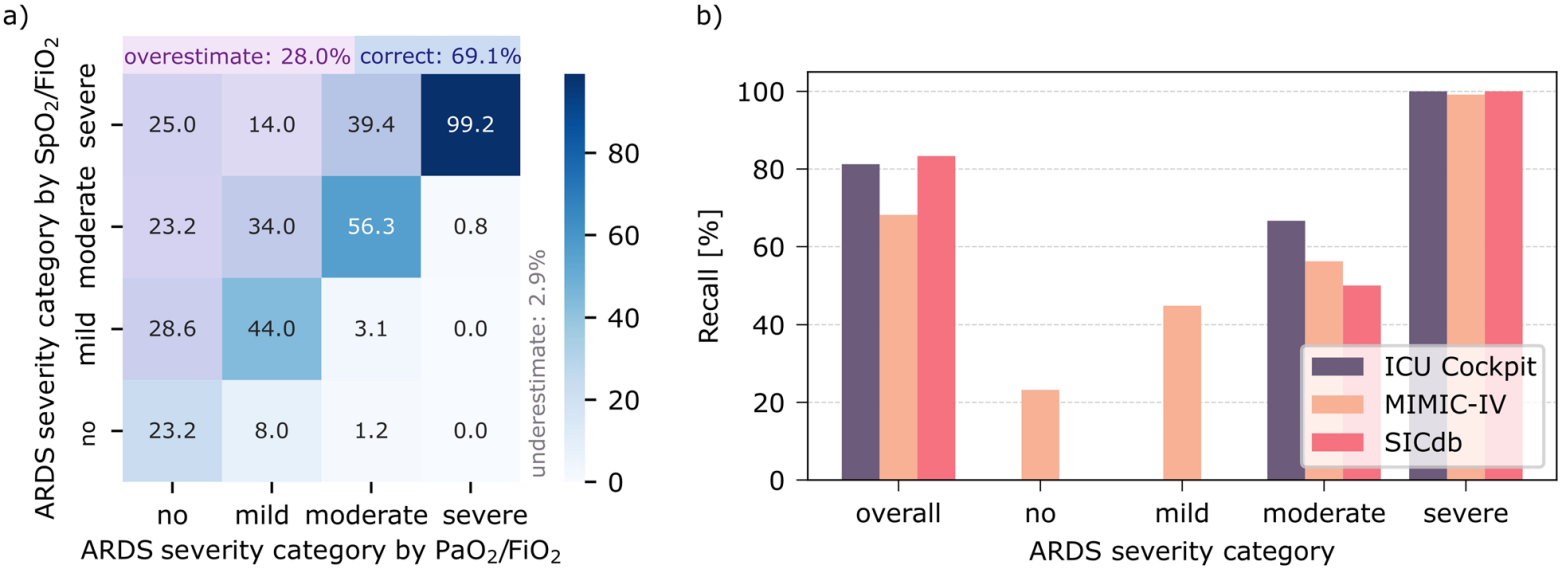
Performance of the SpO_2_/FiO_2_-ratio for ARDS severity classification evaluated on admission level and presented as **a** confusion matrix and **b** recall (sensitivity) per ARDS category. Numbers are presented as percentages of PaO_2_/FiO_2_ category

Alignment of ARDS severity categories was 69.1% comparing ARDS severity per admission (Fig. 1a), and 67.1% considering individual datapoints (see Supplementary Information Fig. S4a). Results were similar in all databases (Fig. S5). Performance was best in more severe PaO_2_/FiO_2_ categories (Fig. 1b, Supplementary Information Fig. S4b). The SpO_2_/FiO_2_-ratio overestimated the PaO_2_/FiO_2_-ratio category in 28.0% and underestimated it in 2.9% of admissions. Accuracy differed between databases with high time-resolution (SpO2 measured every second in ICU Cockpit, and every minute in SICdb) and MIMIC-IV, which has lower granularity of one hour (Fig. 1b and Supplementary Information Fig. S4b).

### Influence of FiO_2_ settings

**Fig. 2:**
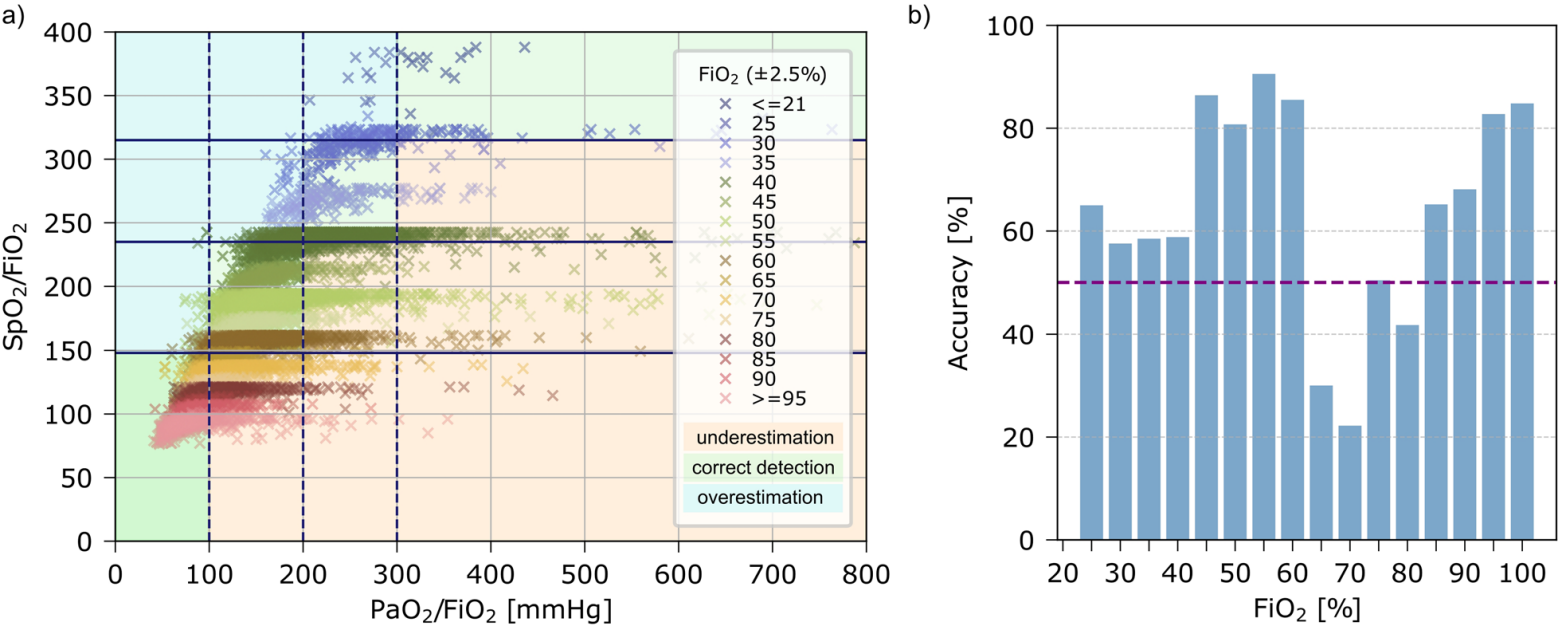
Impact of FiO_2_ on accuracy of SpO_2_/FiO_2_-ratio. **a** PaO_2_/FiO_2_ vs. SpO_2_/FiO_2_ colored by FiO_2._ Lines represent thresholds of SpO_2_/FiO_2_ (solid) and PaO_2_/FiO_2_ (dashed). **b** ARDS severity classification accuracy in respect to FiO_2._ Dashed line marks an accuracy of 50%

A moderate/good correlation was found between SpO_2_/FiO_2_ and PaO_2_/FiO_2_ (r = 0.69). SpO_2_/FiO_2_-ratio was highly influenced by FiO_2_ setting, visualized by separation of ARDS severity categories by FiO_2_ (Fig. 2a). Similar, FiO_2_ settings were more widely distributed using the PaO_2_/FiO_2_-ratio and more distinct using the SpO_2_/FiO_2_-ratio categories (see Supplementary Information Fig. S6). Classification accuracy also differs by FiO_2_ settings, ranging from an accuracy of 22% at FiO_2_ 70 (± 2.5)% to an accuracy of 91% at FiO_2_ 55 (± 2.5)% as presented in Fig. 2c.

### Trending ability of the SpO_2_/FiO_2_-ratio

**Fig. 3:**
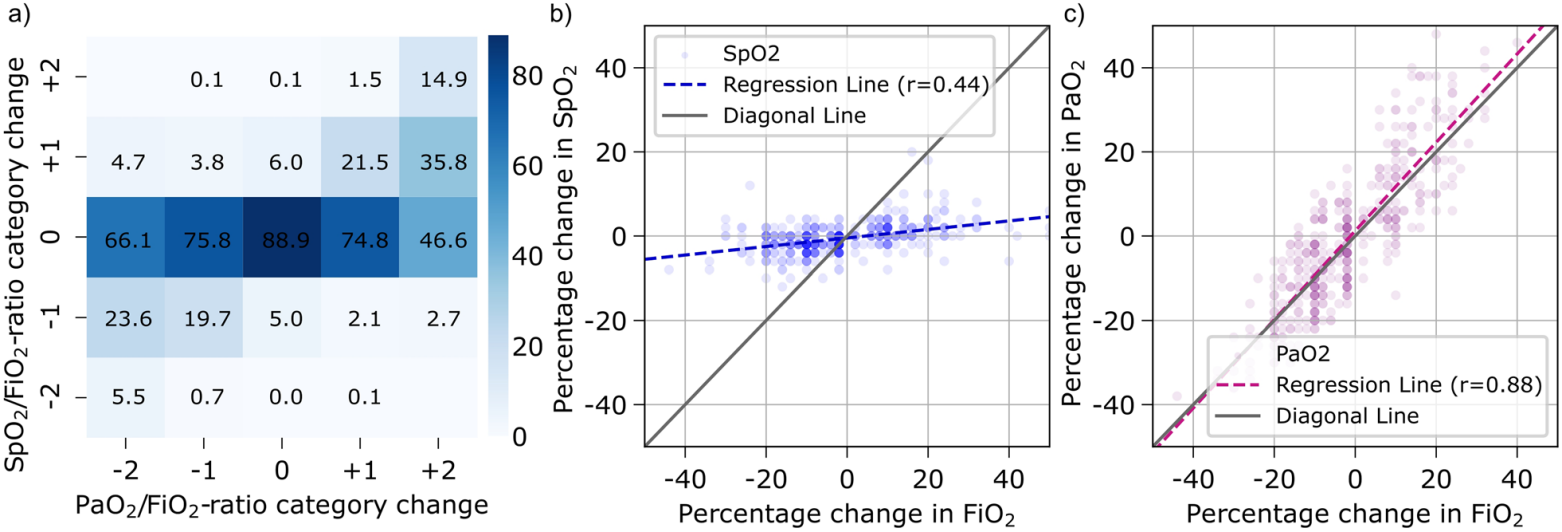
Trending ability of SpO_2_/FiO_2_-ratio. **a** Confusion matrix for changes in ARDS severity category. Proportional change of **b** SpO_2_ and **c** PaO_2_ when FiO_2_ changed during a stable respiratory condition (Respiratory Index ± 20%). Numbers are presented as percentages

The SpO_2_/FiO_2_-ratio correctly detected changes in PaO_2_/FiO_2_-ratios severity categories between two consecutive datapoints in only 19.6% of changes (Fig. 3a).

During relatively stable respiratory conditions (RI within ± 20%), proportional changes in FiO_2_ were strongly correlated with proportional changes in PaO_2_ (r = 0.88), indicating similar PaO_2_/FiO_2_-ratios. These changes in FiO_2_ were not correlated with proportional changes in SpO_2_ (r = 0.44), indicating that FiO_2_ changes alter the SpO_2_/FiO_2_-ratio disproportionately (Fig. 3b and c).

### Limitations of SpO_2_ measurements

**Fig. 4:**
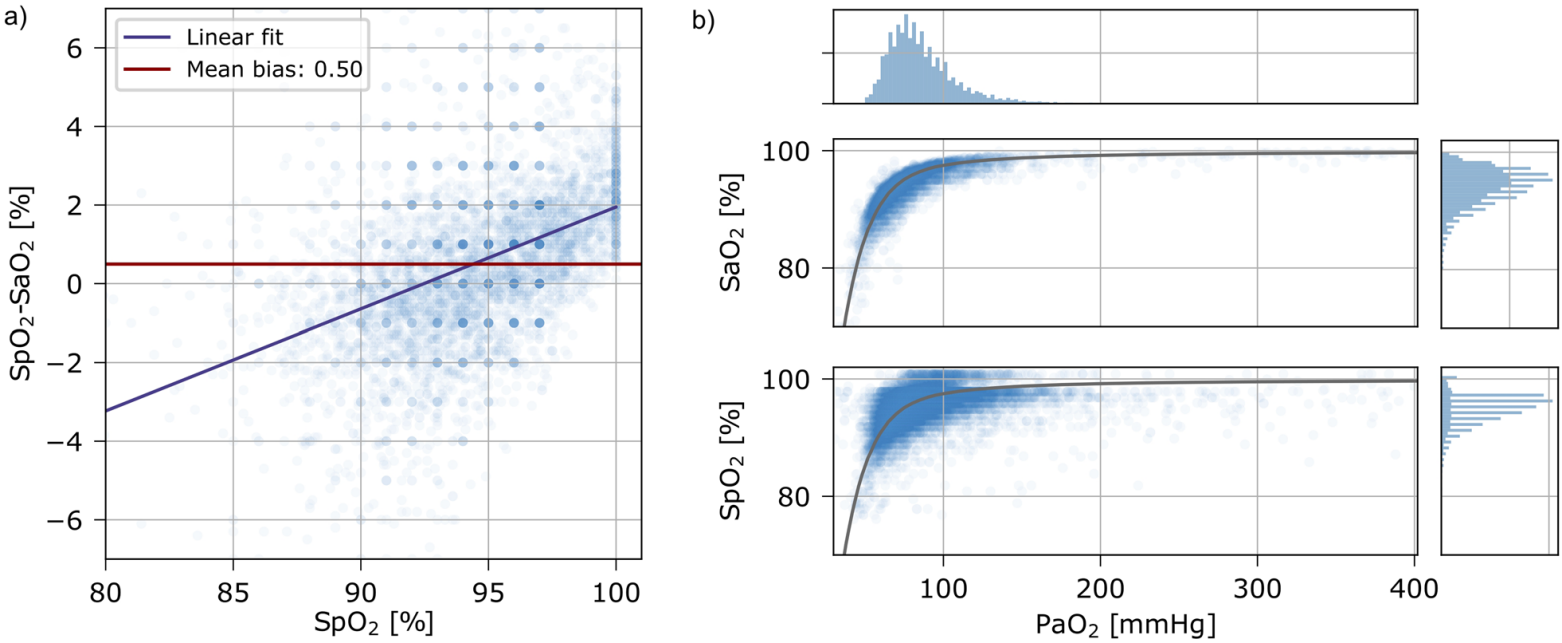
Relationship between SpO_2_, SaO_2_ and PaO_2_. **a** SpO_2_ vs. SpO_2_–SaO_2_. The midhorizontal line marks the mean difference between SpO_2_ and SaO_2_ (bias, + 0.5%). **b** Measured SpO_2_, SaO_2_ and PaO_2_. The grey curve represents the extrapolated oxyhemoglobin dissociation curve by Severinghaus [28]. Histograms for SaO_2_, SpO_2_ (right) and PaO_2_ (top) are shown. SpO_2_ was not restricted to ≤ 97%, but datapoints with a PaO_2_ above 400 mmHg were excluded for visualization

Mean difference (bias) and precision of SpO_2_ measurements compared to SaO_2_ were 0.5 ± 2.1%. Bias showed a heteroscedastic trend towards negative bias below and positive bias above an oxygen saturation of 94% (Fig. 4a). The relationship between PaO_2_ and SaO_2_ can be described by the oxyhemoglobin dissociation curve proposed by Severinghaus [28]. This relationship was diluted for SpO_2_ (Fig. 4b).

### Limitations of conversion from SpO_2_/FiO_2_ to PaO_2_/FiO_2_

**Fig. 5:**
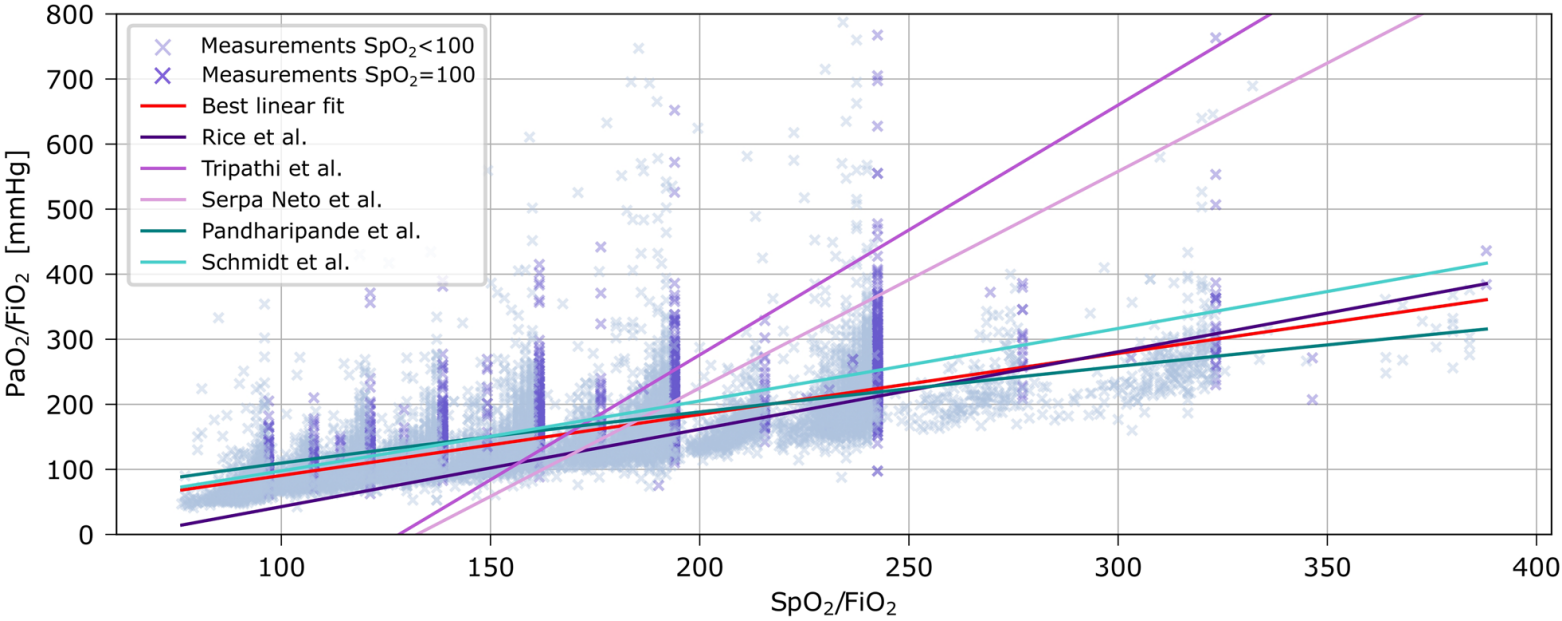
Relationship between PaO_2_/FiO_2_ and SpO_2_/FiO_2_ and comparison of best linear fit with imputations proposed in the literature [10–14]

Out of the linear imputations of PaO_2_/FiO_2_ from SpO_2_/FiO_2_, the equation from Rice et al. [10] best fitted the data, whereas out of the log-linear equations, the one proposed by Pandharipande et al. [13] fitted best. The fits of these equations are presented in Fig. 5. Performance data is available in Table S1.

## Discussion

In this retrospective analysis using data from ARDS patients out of three ICU databases, we showed that classification of ARDS severity by SpO_2_/FiO_2_-ratio leads to misclassification in 33% of datapoints and 31% of admissions. Further, SpO_2_/FiO_2_-ratio showed limited trending ability of disease progression and high dependence on FiO_2_ setting.

The SpO_2_/FiO_2_-ratio classified two thirds of datapoints correctly into ARDS severity categories. Of the misclassified events, the majority was overestimated in severity (84% of the misclassified datapoints). The misclassification rate is strongly influenced by FiO2, with the lowest accuracy observed at a FiO_2_ level of 70%, where application of the SpO_2_/FiO_2_-ratio inevitably assumes severe ARDS, irrespective of SpO_2_. While proportional changes in FiO_2_ let to similar changes in PaO_2_ (r = 0.88), we found that SpO_2_ reacts only poorly (r = 0.42) to changes in FiO_2_. This introduces a major treatment effect, as SpO_2_/FiO_2_ is determined primarily by FiO_2_ settings rather than measured SpO_2_. Changing the FiO_2_ to a higher value will most likely lead to a lower SpO_2_/FiO_2_-ratio despite unchanged gas exchange conditions. This hinders any comparison of patients with different FiO_2_ or even the same patient at different time points.

The PaO_2_/FiO_2_ ratio, is also not a perfect surrogate of pulmonary oxygen gas transfer as it is not reliably reflecting intrapulmonary shunt [29], is not independent from FiO_2_ [30] and influenced by extrapulmonary factors like hemoglobin [7]. Furthermore, PaO_2_/FiO_2_-ratio is not stable during transition from HFNO or NIV to mechanical ventilation [31]. Nevertheless the PaO_2_/FiO_2_-ratio showed better stability over FiO_2_ changes compared to the SpO_2_/FiO_2_-ratio [32]. While RI shares some of these limitations, it was shown to correlate best with intrapulmonary shunt out of all tension-based indices of oxygenation [29] and has less variability over the range of FiO_2_ compared to the PaO_2_/FiO_2_ ratio [27]. For evaluating the influence of FiO_2_ changes, we therefore chose a stable Respiratory Index (± 20%) as a prerequisite to exclude large changes in pulmonary oxygen transfer capability, which would make interpretation difficult.

PaO_2_/FiO_2_ has been used extensively to track disease progression and response to interventions [5]. A continuous estimate of pulmonary gas transfer may enhance detection of clinical worsening and would fill the gaps between intermittent ABGs for more complex machine learning algorithms. Our data showed, that the SpO_2_/FiO_2_-ratio captures severity category changes correctly only in about 20% of events when PaO_2_/FiO_2_ severity category changed. As SpO_2_/FiO_2_ primarily reacts on treatment changes (FiO_2_) which probably occur after worsening oxygenation was detected on ABG, the SpO_2_/FiO_2_-ratio has limited use for monitoring of disease.

While the accuracy of pulse oximetry may be reasonable for avoiding hypoxemia [33], SpO_2_ may overestimate [34] or underestimate [35] arterial oxygen saturation, which probably depends on the type of oximeter [36, 37] and skin pigmentation [38]. We found a heteroscedastic bias for SpO_2_ of + 0.5% compared to SaO_2_, resulting in negative bias below and positive bias above 94%. A precision of ± 2.2% in our data is in line with other studies [36, 39], some of which also found a heteroscedastic bias for SpO_2_ [35]. SpO_2_ measurements are prone to artifacts, mainly low-perfusion states and movement artifacts [40], which were minimized in this analysis by using the median SpO_2_ over a short time interval. In clinical practice this means, that artifacts need to be carefully excluded before drawing assumptions based on SpO_2_ and device internal computations may differ between centers.

Oximetry is relatively insensitive in detection of oxygenation in patient with high PaO_2_ [41], because of the sigmoid shape of the oxyhemoglobin dissociation curve. Partial pressure of carbon dioxide, pH and temperature are known to shift this curve and therefore lead to imprecision [42]. However, SaO_2_ seem to follow the data from the Severinghaus Eq. (28) quite well, suggesting that the main reason for using a linear approach for converting SpO_2_/FiO_2_ to PaO_2_/FiO_2_ may be the missing precision of SpO_2_. Indeed, in our data, SpO_2_ measurements ≤ 97% followed best a linear equation.

Comparing other equations for conversion of SpO_2_/FiO_2_ to PaO_2_/FiO_2_ [10–14], the equation from Rice et al. [10], fitted the data better than other linear imputations. Non-linear approaches may fit the data even better, but are not easily calculated at bedside. A different equation or other thresholds for ARDS severity categories may marginally improve discrimination, but this does not solve the problem of the overweighed effect of FiO_2_. Rescaling (e.g. multiplying SpO_2_ in the equation) may be an option, but bears the risk of inflating imprecision of SpO_2_ measurements.

This study has some limitations, including the retrospective design, which may lead to selection bias. To minimize this risk, we included an extended population from MIMIC-IV, where patients were manually selected based on ARDS criteria and achieved similar results. The resolution of time data such as SpO_2_ in MIMIC-IV hinders perfect matching of SpO_2_ and PaO_2_ values and may introduced inaccuracy. We therefore chose to include three different databases, despite unequal distribution of patients. The analysis is still most affected by MIMIC-IV, which has the highest number of patients. Results remain similar when restricting the analysis to the ICU Cockpit or SICdb, which cover data with high time-resolution and allow matching of the exact ABG time point by analyzing the gap in invasive blood pressure readings (ICU Cockpit). Only a slightly better accuracy, however was found for high-resolution data. We therefore consider the results generalizable to other centers. We did not collect outcome data, because classification based on SpO_2_/FiO_2_ was not established in high resource areas, where regular ABG measurements are available. A retrospective outcome comparison would therefore ignore treatment effects. Future research should compare outcomes of patients in settings where the new global definition is in use. Regarding this study, we do not know if classification discrepancies are associated with harm or cause differences in treatment such as the application of lung protective ventilation, time of intubation, allowing spontaneous breathing efforts or changes in resource allocation. Finally, the analysis did not differentiate between HFNO and mechanical ventilation. It remains an open question whether the performance differs between the two modes of respiratory support.

In summary, while the SpO_2_/FiO_2_ ratio seems to discriminate ARDS patients into categories that have similar mortality compared to the PaO_2_/FiO_2_ ratio [16, 17], and has relatively good correlation with PaO_2_/FiO_2_ (r = 0.69), we showed that these two indices should not be used interchangeably. In resource-limited settings without the ability to draw ABGs, we suggest creating standardized settings, e.g. avoid movement artifacts, and optimize perfusion. Further, due to strong impact of FiO_2_, we suggest minimizing FiO_2_, so that SpO_2_ is at the lower limit of target range. However, as target SpO_2_ range may vary across centers, due to conflicting evidence of a best target range [43–47], ARDS severity classification may still not be comparable. Alternatively, better performance using non-invasive approaches to classify ARDS may be achieved through more complex machine learning models.

## Conclusion

The use of the SpO_2_/FiO_2_-ratio for severity classification in ARDS interchangeably with the PaO_2_/FiO_2_-ratio is limited by its high dependence on FiO_2_ settings, the inability to follow disease progression and the imprecision of SpO_2_ measurements. This discrepancy in severity classification may influence treatment decisions and patient selection in clinical trials.

## Supplementary Information

Additional file 1.

## Data Availability

The databases MIMIC-IV and SICdb are freely-accessible through PhysioNet [23] after training and signing the data use agreement. Retrieval codes for this data are available from the corresponding author on reasonable request. The database ICU Cockpit is not publicly available due to reasons of sensitivity, based on the protocol approved by the Cantonal Ethics Commission of Zurich. Some metadata are published on Zenodo (https://zenodo.org/records/12635089). The study report adheres to the Strengthening the Reporting of Observational Studies in Epidemiology (STROBE) Statement [48].

## Abbreviations

ABG: Arterial blood gas
ARDS: Acute respiratory distress syndrome
CPAP: Continuous positive airway pressure
FiO_2_: Fraction of inspired oxygen
HFNO: High-flow nasal oxygen
ICD: International classification of diseases
ICU: Intensive care unit
MIMIC: Medical information mart for intensive care
NIV: Non-invasive ventilation
PaO_2_: Partial pressure of oxygen in arterial blood
PEEP: Positive end-expiratory pressure
RI: Respiratory index
SaO_2_: Arterial oxygen saturation
SpO_2_: Peripheral oxygen saturation
STROBE: Strengthening the reporting of observational studies in epidemiology

## References

[1] Murray JF, Matthay MA, Luce JM, Flick MR. An expanded definition of the adult respiratory distress syndrome. Am Rev Respir Dis. 1988;138(3):720–3. 3202424 10.1164/ajrccm/138.3.720

[2] Bernard GR, Artigas A, Brigham KL, Carlet J, Falke K, Hudson L, et al. Report of the American-European consensus conference on ARDS: definitions, mechanisms, relevant outcomes and clinical trial coordination. Consens Comm Intensive Care Med. 1994;20(3):225–32. 10.1007/BF01704707 8014293

[3] Force ADT, Ranieri VM, Rubenfeld GD, Thompson BT, Ferguson ND, Caldwell E, et al. Acute respiratory distress syndrome: the berlin definition. JAMA. 2012;307(23):2526–33. 22797452 10.1001/jama.2012.5669

[4] Combes A, Hajage D, Capellier G, Demoule A, Lavoue S, Guervilly C, et al. Extracorporeal Membrane Oxygenation for Severe Acute Respiratory Distress Syndrome. N Engl J Med. 2018;378(21):1965–75. 29791822 10.1056/NEJMoa1800385

[5] Guerin C, Reignier J, Richard JC, Beuret P, Gacouin A, Boulain T, et al. Prone positioning in severe acute respiratory distress syndrome. N Engl J Med. 2013;368(23):2159–68. 23688302 10.1056/NEJMoa1214103

[6] Brower RG, Matthay MA, Morris A, Schoenfeld D, Thompson BT, Wheeler A. Ventilation with lower tidal volumes as compared with traditional tidal volumes for acute lung injury and the acute respiratory distress syndrome. N Engl J Med. 2000;342(18):1301–8. 10793162 10.1056/NEJM200005043421801

[7] Feiner JR, Weiskopf RB. Evaluating pulmonary function: an assessment of PaO2/FIO2. Crit Care Med. 2017;45(1):e40–8. 27618274 10.1097/CCM.0000000000002017

[8] Baskin SB, Oray NC, Yanturali S, Bayram B. The comparison of heparinized insulin syringes and safety-engineered blood gas syringes used in arterial blood gas sampling in the ED setting (randomized controlled study). Am J Emerg Med. 2014;32(5):432–7. 24560392 10.1016/j.ajem.2014.01.020

[9] Riviello ED, Kiviri W, Twagirumugabe T, Mueller A, Banner-Goodspeed VM, Officer L, et al. Hospital incidence and outcomes of the acute respiratory distress syndrome using the kigali modification of the berlin definition. Am J Respir Crit Care Med. 2016;193(1):52–9. 26352116 10.1164/rccm.201503-0584OC

[10] Rice TW, Wheeler AP, Bernard GR, Hayden DL, Schoenfeld DA, Ware LB, et al. Comparison of the SpO2/FIO2 ratio and the PaO2/FIO2 ratio in patients with acute lung injury or ARDS. Chest. 2007;132(2):410–7. 17573487 10.1378/chest.07-0617

[11] Neto AS, Schultz MJ, Festic E, Adhikari NKJ, Dondorp AM, Pattnaik R, et al. Ventilatory support of patients with sepsis or septic shock in resource-limited settings. In: Dondorp AM, Dunser MW, Schultz MJ, editors., et al., Sepsis management in resource-limited settings. Cham: Springer; 2019. p. 131–49. 32091692

[12] Tripathi RS, Blum JM, Rosenberg AL, Tremper KK. Pulse oximetry saturation to fraction inspired oxygen ratio as a measure of hypoxia under general anesthesia and the influence of positive end-expiratory pressure. J Crit Care. 2010;25(3):542.e9-542.e13. 10.1016/j.jcrc.2010.04.009. 20655696 10.1016/j.jcrc.2010.04.009

[13] Pandharipande PP, Shintani AK, Hagerman HE, St Jacques PJ, Rice TW, Sanders NW, et al. Derivation and validation of Spo2/Fio2 ratio to impute for Pao2/Fio2 ratio in the respiratory component of the sequential organ failure assessment score. Crit Care Med. 2009;37(4):1317–21. 19242333 10.1097/CCM.0b013e31819cefa9 PMC3776410

[14] Schmidt MF, Gernand J, Kakarala R. The use of the pulse oximetric saturation to fraction of inspired oxygen ratio in an automated acute respiratory distress syndrome screening tool. J Crit Care. 2015;30(3):486–90. 25746583 10.1016/j.jcrc.2015.02.007

[15] Matthay MA, Arabi Y, Arroliga AC, Bernard G, Bersten AD, Brochard LJ, et al. A new global definition of acute respiratory distress syndrome. Am J Respir Crit Care Med. 2024;209(1):37–47. 37487152 10.1164/rccm.202303-0558WS PMC10870872

[16] Chen W, Janz DR, Shaver CM, Bernard GR, Bastarache JA, Ware LB. Clinical characteristics and outcomes are similar in ARDS diagnosed by oxygen saturation/Fio2 ratio compared with Pao2/Fio2 ratio. Chest. 2015;148(6):1477–83. 26271028 10.1378/chest.15-0169 PMC4665739

[17] Qian F, van den Boom W, See KC. The new global definition of acute respiratory distress syndrome: insights from the MIMIC-IV database. Intensive Care Med. 2024;50(4):608–9. 38483560 10.1007/s00134-024-07383-x

[18] Boss JM, Narula G, Straessle C, Willms J, Azzati J, Brodbeck D, et al. ICU Cockpit: a platform for collecting multimodal waveform data, AI-based computational disease modeling and real-time decision support in the intensive care unit. J Am Med Inform Assoc. 2022;29(7):1286–91. 35552418 10.1093/jamia/ocac064 PMC9196701

[19] Johnson A, Bulgarelli L, Pollard T, Gow B, Moody B, Horng S, et al. MIMIC-IV.

[20] Johnson AEW, Bulgarelli L, Shen L, Gayles A, Shammout A, Horng S, et al. MIMIC-IV, a freely accessible electronic health record dataset. Sci Data. 2023;10(1):1. 36596836 10.1038/s41597-022-01899-x PMC9810617

[21] Rodemund N, Kokoefer, A., Wernly, B., & Cozowicz, C. Salzburg intensive care database (SICdb), a freely accessible intensive care database (version 1.0.6). In: PhysioNet, editor. 2023. 10.1007/s00134-023-07046-3 PMC10287776 37052626

[22] Rodemund N, Wernly B, Jung C, Cozowicz C, Koköfer A. The salzburg intensive care database (SICdb): an openly available critical care dataset. Intensive Care Med. 2023;49(6):700–2. 37052626 10.1007/s00134-023-07046-3 PMC10287776

[23] Goldberger AL, Amaral LA, Glass L, Hausdorff JM, Ivanov PC, Mark RG, et al. PhysioBank, physiotoolkit, and physionet: components of a new research resource for complex physiologic signals. Circulation. 2000;101(23):E215–20. 10851218 10.1161/01.cir.101.23.e215

[24] Bellani G, Laffey JG, Pham T, Fan E, Brochard L, Esteban A, et al. Epidemiology, patterns of care, and mortality for patients with acute respiratory distress syndrome in intensive care units in 50 countries. JAMA. 2016;315(8):788–800. 26903337 10.1001/jama.2016.0291

[25] Laghi F, Siegel JH, Rivkind AI, Chiarla C, DeGaetano A, Blevins S, et al. Respiratory index/pulmonary shunt relationship: quantification of severity and prognosis in the post-traumatic adult respiratory distress syndrome. Crit Care Med. 1989;17(11):1121–8. 2791591

[26] Helmholz HF Jr. The abbreviated alveolar air equation. Chest. 1979;75(6):748. 436542 10.1378/chest.75.6.748

[27] Kathirgamanathan A, McCahon RA, Hardman JG. Indices of pulmonary oxygenation in pathological lung states: an investigation using high-fidelity, computational modelling. Br J Anaesth. 2009;103(2):291–7. 19541678 10.1093/bja/aep140

[28] Severinghaus JW. Simple, accurate equations for human blood O2 dissociation computations. J Appl Physiol Respir Environ Exerc Physiol. 1979;46(3):599–602. 35496 10.1152/jappl.1979.46.3.599

[29] Cane RD, Shapiro BA, Templin R, Walther K. Unreliability of oxygen tension-based indices in reflecting intrapulmonary shunting in critically ill patients. Crit Care Med. 1988;16(12):1243–5. 3191742 10.1097/00003246-198812000-00014

[30] Gattinoni L, Vassalli F, Romitti F. Benefits and risks of the P/F approach. Intensive Care Med. 2018;44(12):2245–7. 30353385 10.1007/s00134-018-5413-4

[31] Ranieri VM, Tonetti T, Navalesi P, Nava S, Antonelli M, Pesenti A, et al. High-flow nasal oxygen for severe hypoxemia: oxygenation response and outcome in patients with COVID-19. Am J Respir Crit Care Med. 2022;205(4):431–9. 34861135 10.1164/rccm.202109-2163OC PMC8886947

[32] Shen Y, Zhu L, Yan J. Stability of Spo2/Fio2 and respiratory rate-oxygenation indexes in critical respiratory disorders. Crit Care Med. 2022;50(8):e694–5. 35838269 10.1097/CCM.0000000000005559

[33] Jubran A. Pulse oximetry. Critical Care. 2015;19(1):272. 26179876 10.1186/s13054-015-0984-8 PMC4504215

[34] Seguin P, Le Rouzo A, Tanguy M, Guillou YM, Feuillu A, Malledant Y. Evidence for the need of bedside accuracy of pulse oximetry in an intensive care unit. Crit Care Med. 2000;28(3):703–6. 10752818 10.1097/00003246-200003000-00017

[35] Perkins GD, McAuley DF, Giles S, Routledge H, Gao F. Do changes in pulse oximeter oxygen saturation predict equivalent changes in arterial oxygen saturation? Critical Care. 2003;7(4):R67. 12930558 10.1186/cc2339 PMC270702

[36] Van de Louw A, Cracco C, Cerf C, Harf A, Duvaldestin P, Lemaire F, Brochard L. Accuracy of pulse oximetry in the intensive care unit. Intensive Care Med. 2001;27(10):1606–13. 11685301 10.1007/s001340101064

[37] Blanchet MA, Mercier G, Delobel A, Nayet E, Bouchard PA, Simard S, et al. Accuracy of multiple pulse oximeters in stable critically Ill patients. Respir Care. 2023;68(5):565–74. 36596654 10.4187/respcare.10582 PMC10171338

[38] Jubran A, Tobin MJ. Reliability of pulse oximetry in titrating supplemental oxygen therapy in ventilator-dependent patients. Chest. 1990;97(6):1420–5. 2347228 10.1378/chest.97.6.1420

[39] Webb RK, Ralston AC, Runciman WB. Potential errors in pulse oximetry: II. Effects of changes in saturation and signal quality. Anaesthesia. 1991;46(3):207–12. 2014899 10.1111/j.1365-2044.1991.tb09411.x

[40] Clayton DG, Webb RK, Ralston AC, Duthie D, Runciman WB. A comparison of the performance of 20 pulse oximeters under conditions of poor perfusion. Anaesthesia. 2007;46(1):3–10. 10.1111/j.1365-2044.1991.tb09303.x 1996749

[41] Jubran A. Pulse oximetry. Critical Care. 1999;3(2):R11–7. 11094477 10.1186/cc341 PMC137227

[42] Collins JA, Rudenski A, Gibson J, Howard L, O’Driscoll R. Relating oxygen partial pressure, saturation and content: the haemoglobin-oxygen dissociation curve. Breathe. 2015;11(3):194–201. 26632351 10.1183/20734735.001415 PMC4666443

[43] Barrot L, Asfar P, Mauny F, Winiszewski H, Montini F, Badie J, et al. Liberal or conservative oxygen therapy for acute respiratory distress syndrome. N Engl J Med. 2020;382(11):999–1008. 32160661 10.1056/NEJMoa1916431

[44] Mackle D, Bellomo R, Bailey M, Beasley R, Deane A, Eastwood G, Finfer S, Freebairn R, King V, Linke N, Litton E. Conservative oxygen therapy during mechanical ventilation in the ICU. New England J Med. 2019;382(11):989–98. 31613432 10.1056/NEJMoa1903297

[45] Schjorring OL, Klitgaard TL, Perner A, Wetterslev J, Lange T, Siegemund M, et al. Lower or higher oxygenation targets for acute hypoxemic respiratory failure. N Engl J Med. 2021;384(14):1301–11. 33471452 10.1056/NEJMoa2032510

[46] Semler MW, Casey JD, Lloyd BD, Hastings PG, Hays MA, Stollings JL, et al. Oxygen-saturation targets for critically Ill adults receiving mechanical ventilation. N Engl J Med. 2022;387(19):1759–69. 36278971 10.1056/NEJMoa2208415 PMC9724830

[47] Girardis M, Busani S, Damiani E, Donati A, Rinaldi L, Marudi A, et al. Effect of conservative vs conventional oxygen therapy on mortality among patients in an intensive care unit: the oxygen-ICU randomized clinical trial. JAMA. 2016;316(15):1583–9. 27706466 10.1001/jama.2016.11993

[48] von Elm E, Altman DG, Egger M, Pocock SJ, Gotzsche PC, Vandenbroucke JP, Initiative S. The strengthening the reporting of observational studies in epidemiology (STROBE) statement: guidelines for reporting observational studies. Lancet. 2007;370(9596):1453–7. 18064739 10.1016/S0140-6736(07)61602-X

