# Supplementary material for: Limitations of SpO2 / FiO2-ratio for classification and monitoring of acute respiratory distress syndrome—an observational cohort study
Source: Crit Care. 2025 Feb 19;29:82. doi: 10.1186/s13054-025-05317-7 (PMC11837723; doi:10.1186/s13054-025-05317-7)
Supplement: Supplementary file 1 — Additional file 1. [file 13054_2025_5317_MOESM1_ESM.docx]

Supplementary Information to

Limitations of SpO2 / FiO2 - Ratio for Classification and Monitoring of Acute Respiratory Distress Syndrome: an observational cohort study

Rolf Erlebach^1^*, Una Pale^2^*, Tilman Beck^2^, Sasa Markovic^2^, Marko Seric^2^, Sascha David^1^, Emanuela Keller^2^

1 Institute of Intensive Care Medicine, University Hospital Zurich and University of Zurich, Zurich, Switzerland

2 Neurocritical Care Unit, Department of Neurosurgery and Institute of Intensive Care Medicine, University Hospital and University of Zurich, Zurich, Switzerland

Correspondance to: rolf.erlebach@usz.ch

Contents

[1 Flowcharts of the selection process 3](#_Toc187560489)

[1.1 Main population 3](#_Toc187560490)

[1.2 Extended MIMIC-IV Population 5](#_Toc187560491)

[2 Supplemental Figures of the Main Population 6](#_Toc187560492)

[2.1 ARDS severity distribution in different databases 6](#_Toc187560493)

[2.2 Clinical performance of the SpO_2_/FiO_2_-ratio 7](#_Toc187560494)

[2.3 Clinical performance in individual databases 8](#_Toc187560495)

[2.4 Influence of FiO_2_ settings 9](#_Toc187560496)

[3 Performance metrics of proposed imputations of PaO_2_/FiO_2_ from SpO_2_/FiO_2_ 10](#_Toc187560497)

[4 Supplemental Figures of the extended MIMIC-IV Population 11](#_Toc187560498)

[4.1 Clinical performance of the SpO_2_/FiO_2_-ratio 11](#_Toc187560499)

[4.2 Influence of FiO_2_ settings 12](#_Toc187560500)

[4.3 Trending ability of the SpO_2_/FiO_2_-ratio 13](#_Toc187560501)

[5 References 14](#_Toc187560502)

# Flowcharts of the selection process

## Main population


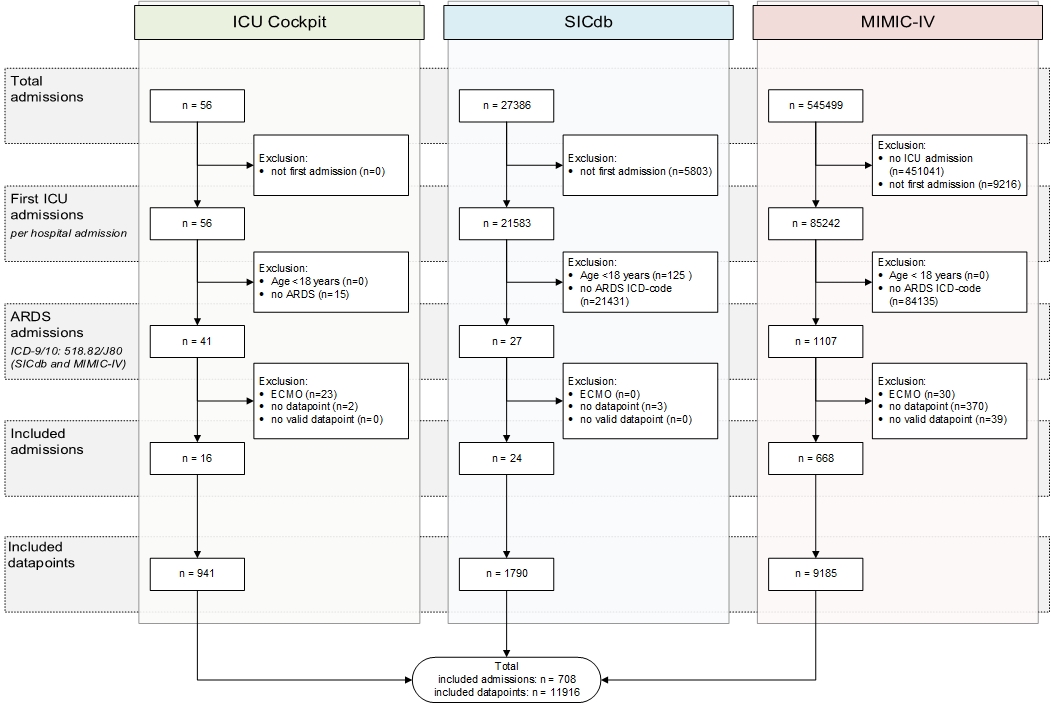


**Fig. S1** Flowchart of patient selection for the main population. The ICD codes used for MIMIC-IV and SICdb patient selection were: 518.82 ( ICD-9) and J80 (ICD-10). ARDS definition in the ICU Cockpit database followed the Berlin definition, but the hypoxemia criterion was modified to include patients with PaO_2_/FiO_2_ ≤300 mmHg measured during invasive mechanical ventilation, NIV or CPAP with PEEP ≥5 cmH_2_O or during HFNO with flow ≥30 L/min. Datapoints represent time-matched sets of SpO_2_, FiO_2_ and PaO_2_. Datapoints were valid if SpO_2_ was ≤97% and measurements were taken during invasive mechanical ventilation, NIV or CPAP with PEEP ≥5 cmH_2_O or HFNO with flow ≥30 L/min. ECMO: Extracorporeal membrane oxygenation.

## Extended MIMIC-IV Population


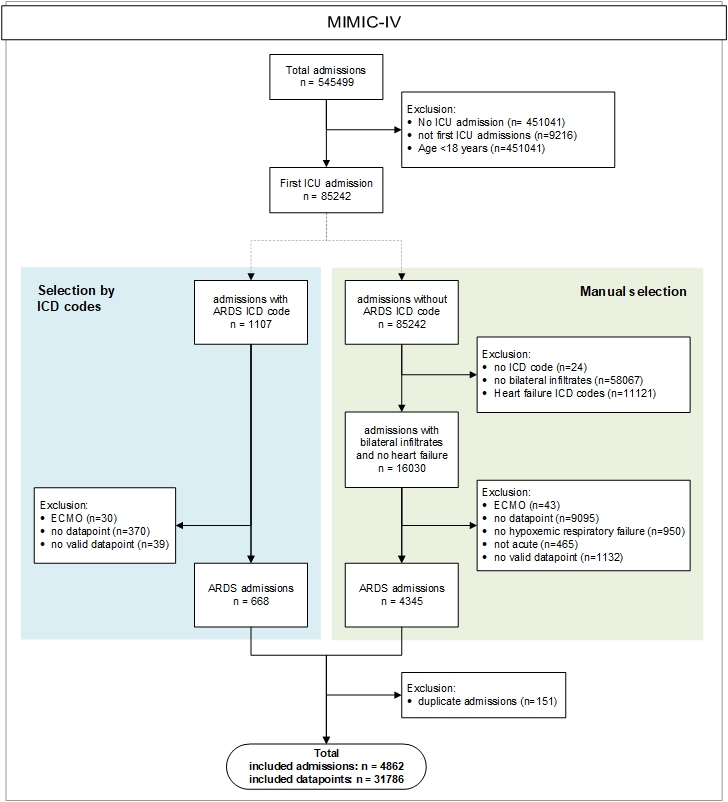


**Fig. S2** Flowchart of patient selection for the extended MIMIC-IV population. The ICD codes used were: 518.82 (ICD-9) and J80 (ICD-10). Datapoints represent time-matched sets of SpO_2_, FiO_2_ and PaO_2_. Datapoints were valid if SpO_2_ was ≤97% and measurements were taken during invasive mechanical ventilation/NIV/CPAP with PEEP ≥5 cmH_2_O or HFNO with flow ≥30 L/min. Hypoxemic respiratory failure was defined as PaO_2_/FiO_2_ ≤300 mmHg, which was considered acute if occurring within 7 days of ICU admission. ECMO: Extracorporeal membrane oxygenation.

# Supplemental Figures of the Main Population

## ARDS severity distribution in different databases

**
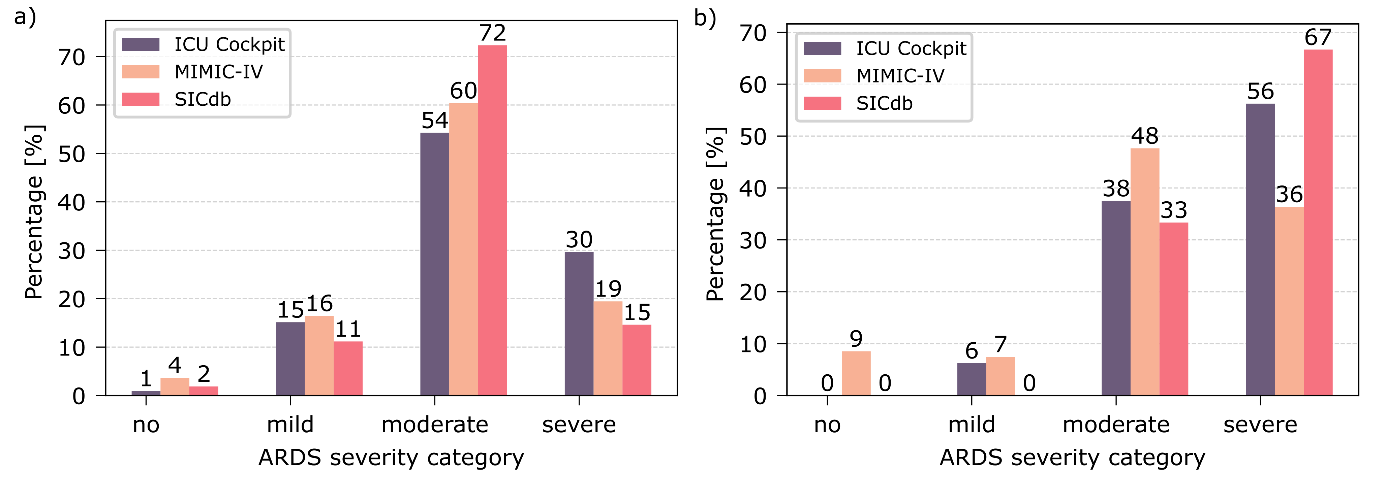
**

**Fig. S3** Distribution of a) datapoints and b) admissions per ARDS severity category for the three databases (MIMIC IV, SICdb and ICU Cockpit).

## Clinical performance of the SpO_2_/FiO_2_-ratio

**
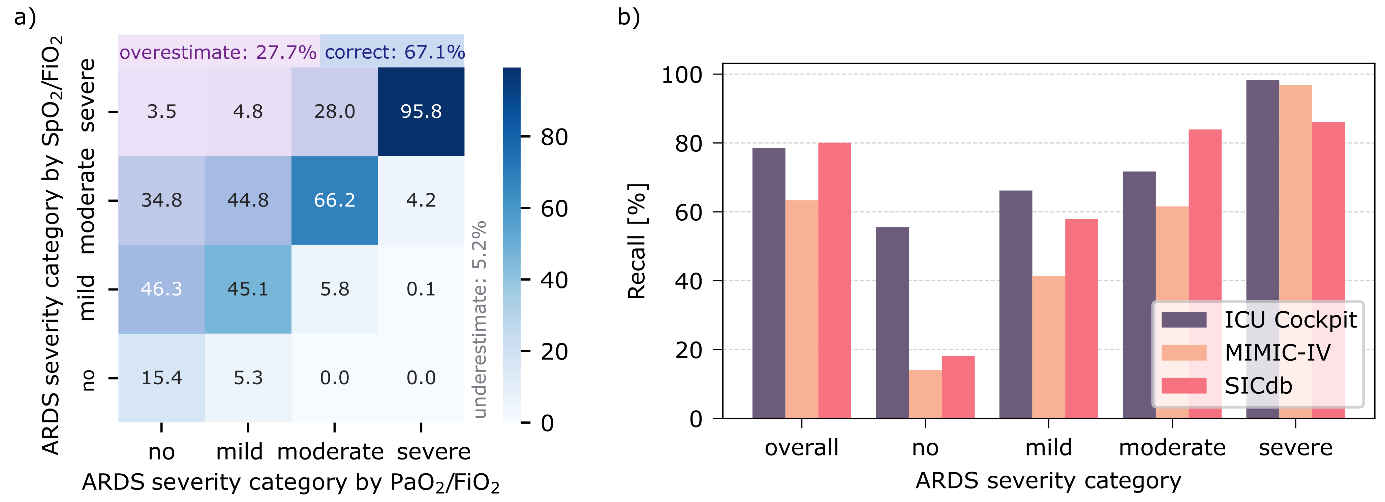
**

**Fig. S4** Performance of the SpO_2_/FiO_2_-ratio for ARDS severity classification evaluated on datapoint level and presented as (a) confusion matrix and (b) recall (sensitivity) per ARDS category. Numbers are presented as percentages of PaO_2_/FiO_2_ category.

## Clinical performance in individual databases


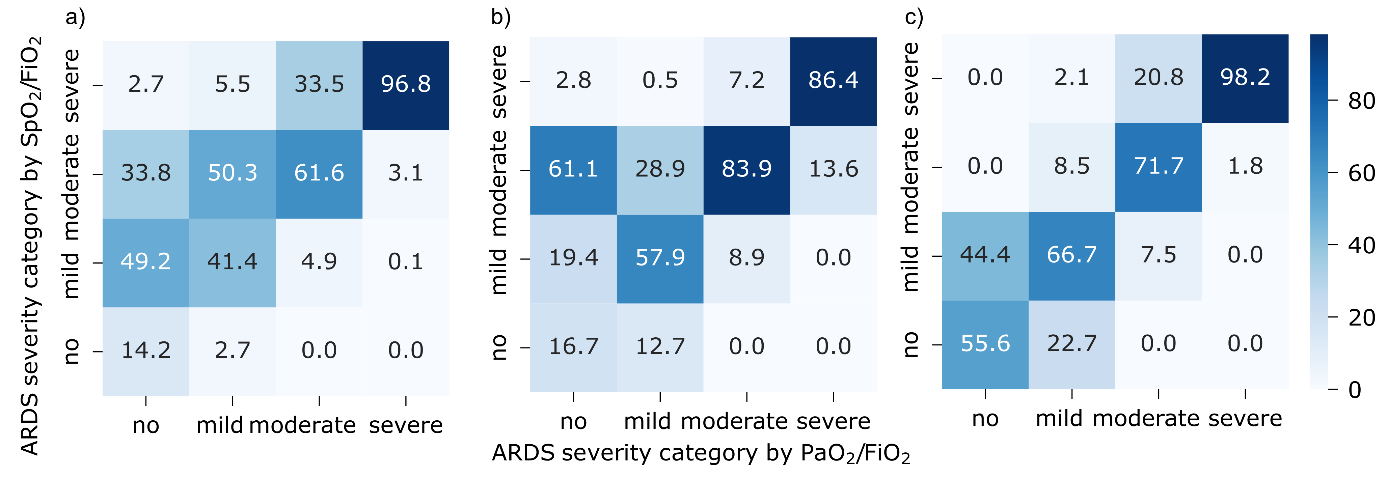


**Fig. S5** Performance of the SpO_2_/FiO_2_-ratio for ARDS severity classification in individual databases evaluated on datapoint level for a) MIMIC-IV, b) SICdb and c) ICU Cockpit. Numbers are presented as percentages of PaO_2_/FiO_2_ category

## Influence of FiO_2_ settings

**
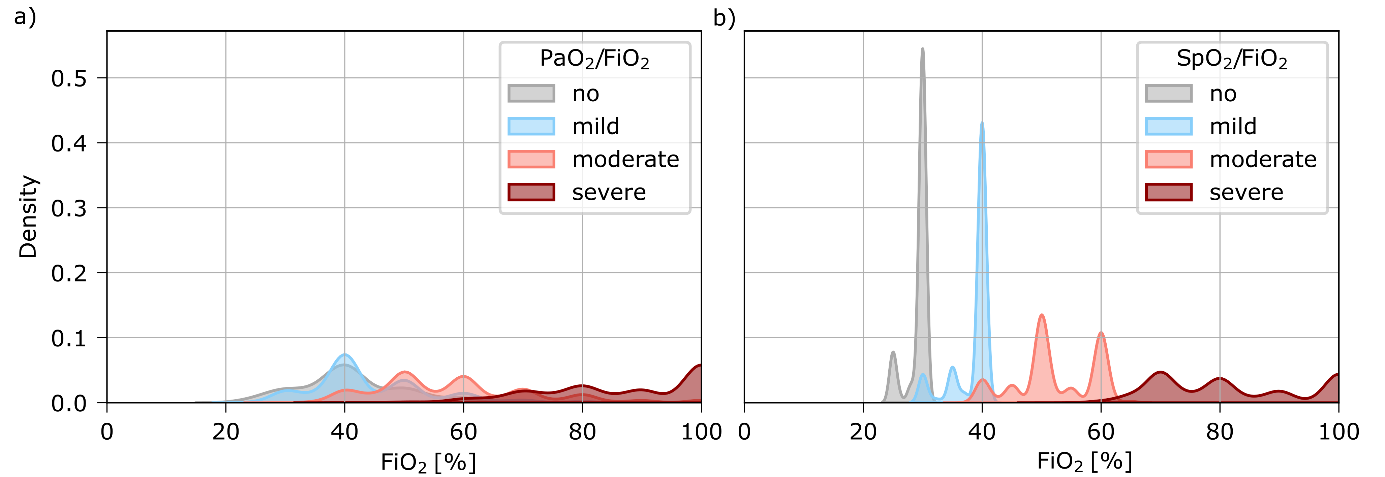
Fig. S6** Density plots by FiO_2_ value for a) PaO_2_/FiO_2_ and b) SpO_2_/FiO_2_ based ARDS severity.

# Performance metrics of proposed imputations of PaO_2_/FiO_2_ from SpO_2_/FiO_2_

| Formula | Type | R^2^ | MAE |
| --- | --- | --- | --- |
| *Best linear fit*  PF = 0.938*SF + -3.236 | Linear | 0,477909284 | 30,76491039 |
| *Rice et al. [1]*  PF = 1.190*SF + -76.300 | Linear | 0,254991306 | 40,31967346 |
| *Tripathi et al. [2]*  PF = 3.840*SF + -492.000 | Linear | -4,092338386 | 129,1018615 |
| *Serpa Neto et al. [3]*  PF = 3.330*SF + -441.000 | Linear | -2,871751612 | 113,7138947 |
| *Pandharipande et al. [4]*  log10(PF) = 0.780*log10(SF) + 0.480 | Log Linear | 0,445097125 | 34,7973141 |
| *Schmidt et al. [5]*  log10(PF) = 1.070*log10(SF) + -0.150 | Log Linear | 0,410890338 | 37,744418 |

**Table S1** Performance metrics of published imputations of PaO_2_/FiO_2_ from SpO_2_/FiO_2_ and the best linear fit of current data. PF: PaO_2_/FiO_2,_ MAE: mean absolute error, SF: SpO_2_/FiO_2._

# ****Supplemental Figures of the extended MIMIC-IV Population****

## Clinical performance of the SpO_2_/FiO_2_-ratio

**
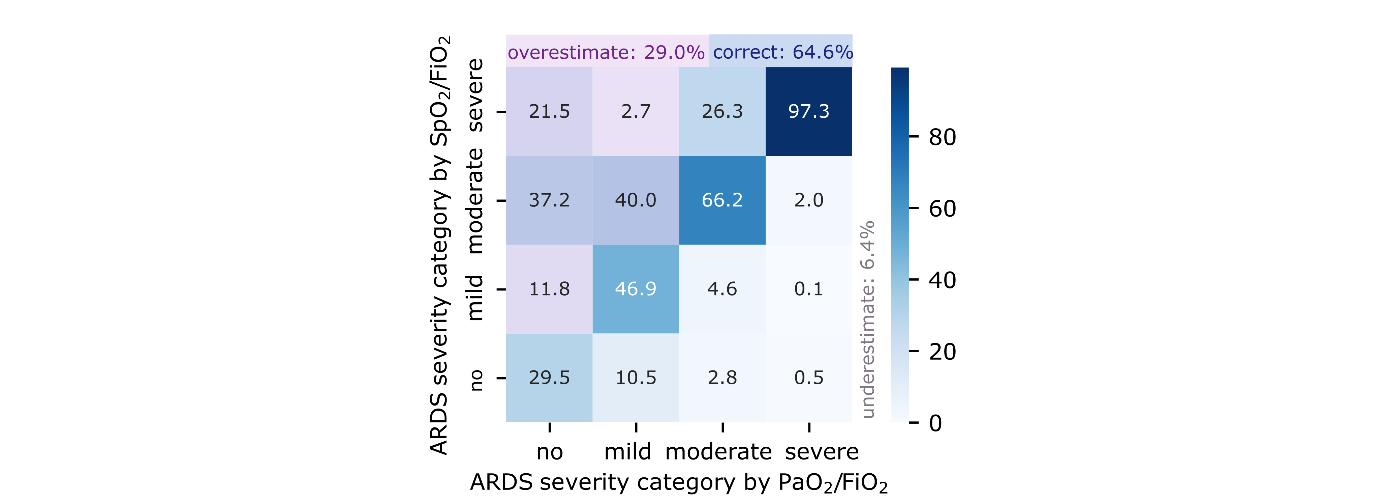
**

**Fig. S****7** Performance of the SpO_2_/FiO_2_-ratio for ARDS severity classification evaluated in the extended MIMIC-IV population on admission level. Numbers are presented as percentages of PaO_2_/FiO_2_ category.

## ****Influence of FiO_2_ settings****

**
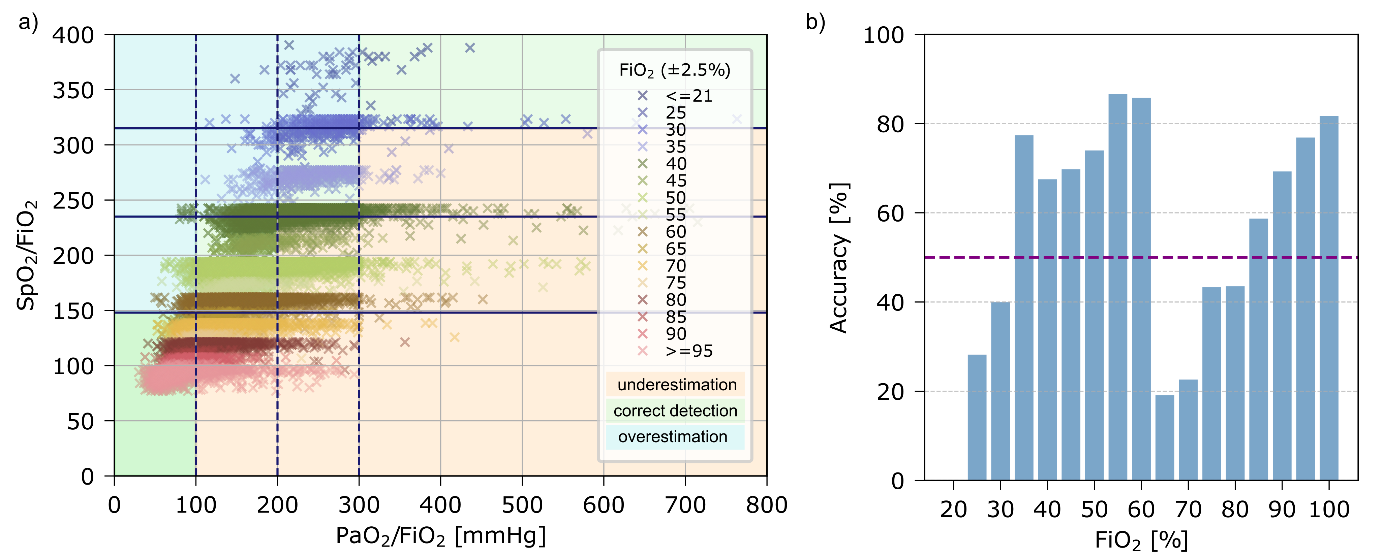
**

**Fig.** **S8** Impact of FiO_2_ on SpO_2_/FiO_2_-ratio and accuracy for ARDS classification in the extended MIMIC-IV population. a) PaO_2_/FiO_2_ vs. SpO_2_/FiO_2_ colored by FiO_2_ value (grouped by 5%). Lines represent thresholds of ARDS severity categories based on SpO_2_/FiO_2_ (solid) and PaO_2_/FiO_2_ (dashed). b) ARDS severity classification accuracy in respect to FiO_2_ value (grouped by 5%). Dashed line marks an accuracy of 50%.

## Trending ability of the SpO_2_/FiO_2_-ratio

**
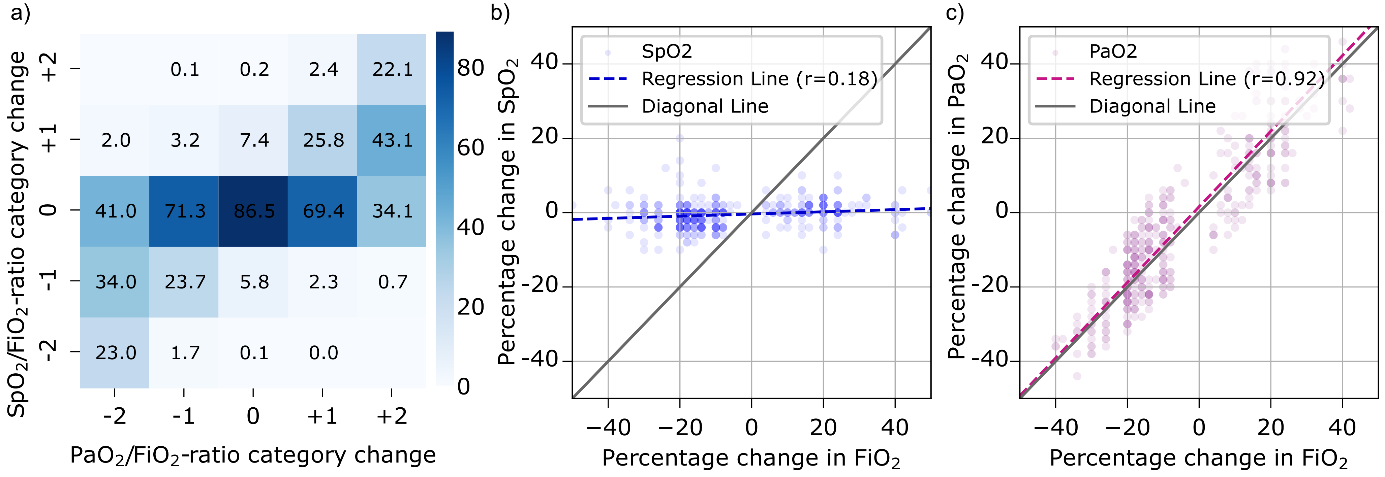
**

**Fig. S9** Performance in the extended MIMIC-IV population of the a) SpO_2_/FiO_2_-ratio to detect changes in ARDS severity classification (confusion matrix) and proportional change of b) SpO_2_ and c) PaO_2_ when FiO_2_ changed during a relatively stable respiratory condition (RI within ± 20%). RI: respiratory index.

# References

1. Rice TW, Wheeler AP, Bernard GR, Hayden DL, Schoenfeld DA, Ware LB, National Institutes of Health NHL, Blood Institute AN, (2007) Comparison of the SpO2/FIO2 ratio and the PaO2/FIO2 ratio in patients with acute lung injury or ARDS. Chest 132: 410-417

2. Tripathi RS, Blum JM, Rosenberg AL, Tremper KK, (2010) Pulse oximetry saturation to fraction inspired oxygen ratio as a measure of hypoxia under general anesthesia and the influence of positive end-expiratory pressure. Journal of critical care 25: 542 e549-513

3. Neto AS, Schultz MJ, Festic E, Adhikari NKJ, Dondorp AM, Pattnaik R, Pisani L, Povoa P, Martin-Loeches I, Thwaites CL (2019) Ventilatory Support of Patients with Sepsis or Septic Shock in Resource-Limited Settings. In: Dondorp AM, Dunser MW, Schultz MJ (eds) Sepsis Management in Resource-limited Settings. Springer Copyright 2019, The Author(s). Cham (CH), pp. 131-149

4. Pandharipande PP, Shintani AK, Hagerman HE, St Jacques PJ, Rice TW, Sanders NW, Ware LB, Bernard GR, Ely EW, (2009) Derivation and validation of Spo2/Fio2 ratio to impute for Pao2/Fio2 ratio in the respiratory component of the Sequential Organ Failure Assessment score. Critical care medicine 37: 1317-1321

5. Schmidt MF, Gernand J, Kakarala R, (2015) The use of the pulse oximetric saturation to fraction of inspired oxygen ratio in an automated acute respiratory distress syndrome screening tool. Journal of critical care 30: 486-490
